# HEART and TIMI Scores Predict Severe Coronary Atherosclerosis in Patients With End-Stage Renal Disease

**DOI:** 10.7759/cureus.40408

**Published:** 2023-06-14

**Authors:** Trishna B Parikh, Moez Aziz, Samuel P Mackoff, Gabriel M Aisenberg

**Affiliations:** 1 Internal Medicine, University of Texas, John P and Kathrine G McGovern School of Medicine, Houston, USA

**Keywords:** dialysis, coronary atherosclerosis, timi score, heart score, end-stage renal disease

## Abstract

Objectives

History, EKG, age, risk factors, and troponin (HEART) and thrombolysis in myocardial infarction (TIMI) risk calculators have been validated to predict the risk of subsequent acute coronary syndromes and in some studies, severe coronary atherosclerosis in patients with a concerning clinical history. Their performance in patients with end-stage renal disease (ESRD), a population with a high pretest probability for the condition, is unknown. We aimed to determine whether HEART and TIMI scores can predict severe coronary atherosclerosis in patients with end-stage renal disease (ESRD).

Methods

A single-center retrospective cohort of admitted patients aged 18 years or older with ESRD on dialysis who underwent coronary angiography during admission (November 2010 to December 2017) was retrospectively reviewed. The outcome of coronary angiography was compared with the calculated HEART and TIMI scores at the time of presentation. Receiver operating characteristics and logistic regression models were used to determine optimal score cutoffs, score usefulness, and associations between outcomes, scores, and patient characteristics.

Results

Among 231 patient encounters, the mean HEART and TIMI scores were 6±2 and 3±1 points, respectively. Patients with diabetes mellitus, those 65 years old and older, and those reported to have angina pectoris were more likely to show severe coronary artery disease (CAD) lesions. Optimal score cutoffs for determining severe coronary lesions were between six and seven (area under the curve (AUC)=0.754, confidence interval (CI): 0.682-0.826) and between three and four (AUC=0.716, CI: 0.640-0.792) for the HEART and TIMI scores, respectively.

Conclusion

Similar to the general population, HEART and TIMI scores can predict severe coronary atherosclerosis in the complex ESRD population.

## Introduction

Cardiovascular disease accounts for 50% of mortality in patients on hemodialysis, with 52% of patients with end-stage renal disease (ESRD) suffering from an acute myocardial infarction within two years of hemodialysis initiation [1,2]. This is attributable to the high prevalence of traditional risk factors such as hypertension and diabetes mellitus and non-traditional risk factors such as vascular calcifications, endothelial dysfunction, and uremia [3-8]. Coronary angiography is the gold standard for diagnosing significant coronary atherosclerosis. While invasive techniques and other imaging modalities can be used to diagnose, risk stratify, and manage coronary artery disease (CAD) in patients with ESRD [4,6,7], acute coronary syndrome (ACS) is sometimes the initial presentation of CAD in this patient population [4]. The HEART and TIMI scores, frequently used to assist in clinical decision-making regarding ACS, use historical, clinical, and laboratory data to predict the likelihood of short-term major adverse cardiac events for patients presenting with non-ST segment elevation myocardial infarction [9-11]. However, the diagnostic evaluation of ACS in the ESRD patient population is a unique clinical challenge because patients may present without chest pain or anginal symptoms and may have abnormal electrocardiogram findings compared to the general population [3,7,8,12].

Though multiple studies have shown positive associations between the HEART and TIMI scores and the angiographic extent of CAD and have been shown to be predictive of clinically significant CAD, none of these studies have specifically addressed patients with ESRD [13-21]. This study aims to determine whether the HEART and TIMI scores are adequate predictors of severe CAD in patients with ESRD.

## Materials and methods

The study was undertaken at Lyndon B. Johnson Hospital, a county-based, safety-net, tertiary care center in Houston, Texas, after obtaining Institutional Review Board approval (HSC-MS-18-0047); patient consent requirements were waived. Using electronic medical records, a retrospective chart review was conducted on encounters dating from November 1, 2010, to December 31, 2017. Patients 18 years of age or older with ESRD on dialysis who were admitted to the hospital and underwent coronary angiogram during that admission were included. Those with incomplete or inaccurate records, ST-segment elevation on electrocardiogram, and outpatients with an elective indication for coronary angiogram were excluded.

From the examined medical records, the following characteristics were evaluated for each patient: age, gender, chest pain reported on presentation, whether that chest pain was interpreted as angina pectoris by the examining provider, a history of diabetes mellitus, hypertension, and hyperlipidemia, obesity, smoking history, family history of early CAD, prior coronary artery bypass graft surgery or percutaneous coronary intervention, a history of peripheral artery disease, stroke, and myocardial infarction, and dialysis schedule. The dialysis schedule was three times weekly or emergent, with the modality adopted among patients with no funding as described elsewhere [22].

We also recorded the highest troponin I level during the event (normal between 0 and 0.045 ng/mL) and description of the electrocardiogram, focusing on the presence of ST-segment depression, non-specific wave abnormalities, T-wave inversions, new Q waves, new right or left bundle branch blocks, and signs of left ventricular hypertrophy. HEART and TIMI scores were calculated based on these findings [9-11]. Coronary angiogram findings were recorded. We defined severe CAD as the presence of any stenotic lesion ≥70% without consideration of the number of affected coronary arteries or left main coronary artery disease. Patients with severe CAD were compared to those with milder or no lesions.

MedCalc Statistical Software version 18 (MedCalc Software, Ostend, Belgium) was utilized for the statistical analysis. Categorical variables were analyzed using the Fisher exact test, and discrete variables were analyzed using the Student t-test for unpaired samples. We calculated a logistic regression model including all the elements upon which the HEART and TIMI scores are built. Adjusted odds ratios (OR) are presented.

To assess the utility of the HEART and TIMI scores in delineating severe CAD, receiver operating characteristic curves were constructed for both scoring systems following existing models [23]. Areas under the curve (AUC) by polynomial regression were calculated for each receiver operating characteristic, and the optimal score cutoffs for each scoring system were determined by measuring the shortest distance from 100% sensitivity and 100% specificity. Confidence intervals were determined assuming a normal distribution. Patients with a history of percutaneous coronary intervention and coronary artery bypass graft were excluded when developing these models. A two-sided p<0.05 was considered indicative of statistical significance.

## Results

Inclusion criteria were met in 231 patient encounters. Baseline characteristics are presented in Table 1. The mean ± standard deviation HEART and TIMI scores were 6±2 and 3±1 points, respectively. In the cohort, 129 (56%) of patients had severe coronary obstructions and 102 (44%) had no or less than severe coronary obstructions. Of those who were found to have severe coronary lesions, 23 (18%) and 34 (26%) had a history of coronary artery bypass graft surgery or percutaneous coronary intervention, respectively.

**Table 1:** Baseline characteristics (n=231 patient encounters) PCI - percutaneous coronary intervention; CABG - coronary-artery bypass graft; CAD - coronary atherosclerotic disease; PAD - peripheral atherosclerotic disease; MI - myocardial infarction, TIMI - thrombolysis in myocardial infarction

Variables	n (%)
Gender (male)	125 (54)
Age (median and range) in years	57 (28 to 82)
Chest pain (unspecified)	138 (60)
Angina pectoris	86 (37)
Previous PCI	48 (21)
Previous CABG	25 (11)
Diabetes mellitus	167 (72)
Hypertension	225 (97)
Hyperlipidemia	51 (22)
Obesity	47 (20)
Smoking	79 (34)
Family history of CAD	38 (16)
History of PAD, stroke, or MI	107 (46)
Aspirin use within 7 days	111 (48)
Scheduled dialysis (3 times/week)	129 (55)
Emergent dialysis	102 (45)
Elevated serum troponin I level	179 (77)
Electrocardiographic abnormalities	
Non-specific T wave abnormalities	129 (56)
New T wave inversion	64 (28)
Left ventricular hypertrophy	60 (26)
ST segment depression	55 (24)
New right bundle branch block	13 (6)
New Q wave	13 (6)
New left bundle branch block	7 (3)
HEART score (average ± standard deviation)	6 ± 2
HEART score more than 6	96 (42)
TIMI score (average ± standard deviation)	3 ± 1
TIMI score more than 3	82 (35)
TIMI score more than 4	42 (18)
Coronary angiogram	
Severe lesions	129 (56)
Less than severe lesions	51 (22)
No lesions	51 (22)
Percutaneous coronary intervention	90 (39)

The indication for obtaining a coronary angiogram was stated by the provider in 224 cases: evaluation of ACS was the most frequent (163 cases), followed by the determination of heart failure etiology (38 cases), elevated serum troponin I levels (11 cases), abnormal stress test (eight cases), and evaluation of aortic stenosis prior to valve replacement (four cases).

A comparison of the characteristics of patients with severe and non-severe or no coronary lesions is presented in Table 2. Patients with severe lesions were older, more frequently complained of angina, had a history of diabetes mellitus, had elevated serum troponin I levels and had ST-segment depression compared to those with non-severe or no lesions. HEART and TIMI scores were significantly higher among patients with severe CAD. Percutaneous coronary intervention was done following coronary angiography in 87 (67%) of patients with severe CAD.

**Table 2:** Comparison of patients with severe coronary lesions and those with less than severe lesions or normal coronary angiograms CAD - coronary atherosclerotic disease; PAD - peripheral atherosclerotic disease; MI - myocardial infarction, TIMI - thrombolysis in myocardial infarction

Variables	Severe lesions, n=129	Less than severe or no lesions, n=102	p-value
Gender (male), n (%)	70 (54)	55 (54)	1.00
Age (mean ± standard deviation)	60 ± 10	52 ± 12	<0.001
Chest pain, n (%)	81 (63)	57 (56)	0.34
Angina, n (%)	56 (43)	30 (29)	0.04
Diabetes mellitus, n (%)	106 (82)	61 (60)	<0.001
Hypertension, n (%)	127 (98)	98 (96)	0.41
Hyperlipidemia, n (%)	31 (24)	20 (20)	0.52
Obesity, n (%)	24 (19)	23 (23)	0.51
Smoking, n (%)	47 (36)	32 (31)	0.48
Family history of CAD, n (%)	22 (17)	16 (16)	0.86
History of PAD, stroke, or MI, n (%)	66 (51)	41 (40)	0.11
Aspirin use within 7 days, n (%)	64 (50)	47 (46)	0.60
Scheduled (versus emergent) dialysis	65 (50)	64 (63)	0.06
Elevated serum troponin I level	108 (84)	69 (68)	0.005
Electrocardiographic abnormalities			
Non-specific T wave abnormalities	72 (56)	57 (56)	1.00
New T wave inversion	36 (28)	28 (27)	1.00
Left ventricular hypertrophy	33 (26)	27 (26)	0.88
ST segment depression	33 (26)	8 (8)	<0.001
New right bundle branch block	10 (8)	4 (4)	0.27
New Q wave	8 (6)	5 (5)	0.78
New left bundle branch block	3 (2)	4 (4)	0.70
HEART score (average ± standard deviation)	7 ± 2	5 ± 2	<0.001
HEART score more than 6	73 (56)	23 (23)	<0.001
TIMI score (average ± standard deviation)	4 ± 1	2 ± 1	<0.001
TIMI score more than 3	61 (47)	21 (21)	<0.001
TIMI score more than 4	36 (28)	6 (6)	<0.001

The logistic regression model found that patients with diabetes mellitus (OR 4.00, 95% confidence interval (CI) 1.80-8.86, p<0.001), those 65 years old and older (OR 3.33, 95% CI 1.40-7.91, p=0.006), and those reported to have angina pectoris (OR 2.55, 95% CI 1.20-5.45, p=0.01) were more likely to show severe CAD lesions.

**Figure 1:**
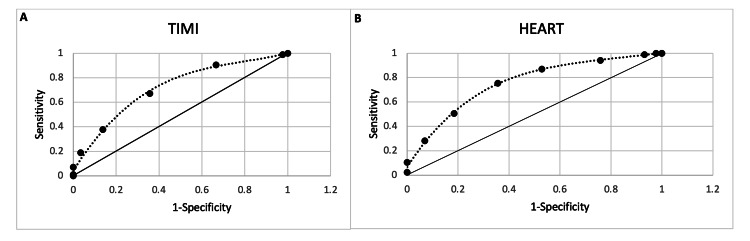
Receiver operating characteristic curves for the TIMI score (A) and HEART score (B), estimated using polynomial regression TIMI - thrombolysis in myocardial infarction

As shown in Figure 1, the optimal score cutoff for delineating severe coronary lesions was between six and seven for the HEART score and between three and four for the TIMI score. The AUC for assessing the overall usefulness of the HEART and TIMI scores in detecting severe coronary artery lesions was 0.754 (CI: 0.682-0.826) and 0.716 (CI: 0.640, 0.792), respectively. Sensitivities for HEART and TIMI scores at optimum are 67.44% and 73.64%, respectively. Specificities for HEART and TIMI scores at optimum are 68.62% and 55.88%, respectively.

## Discussion

Our study demonstrates that a HEART score between six and seven and a TIMI score between three and four are predictive of severe coronary artery lesions in patients suffering from ESRD, with AUCs demonstrating acceptable test utility of 0.754 and 0.716, respectively. The predictors used in calculating the HEART and TIMI scores have been extensively studied in the general population [24-26]; however, the specificity of these predictors may be diminished in the ESRD population [5]. For example, while troponin levels are predictive of cardiovascular events, cardiovascular mortality, and angiographic coronary obstructions in the general population, those levels have limited specificity in patients with ESRD because other cardiac and non-cardiac etiologies contribute to persistent troponin elevation [7-8]. Additionally, left ventricular hypertrophy, anemia, and electrolyte abnormalities are common in this patient population, increasing the likelihood of electrocardiogram abnormalities at baseline [8]. Non-specific electrocardiogram changes, left bundle branch block, and other findings are more prevalent among patients on dialysis with acute myocardial infarction; ST-segment elevation and Q wave changes are more common among non-dialysis patients, while ST-segment depressions are comparable [12]. These differences present a clinical challenge to the physician trying to apply criteria established for the general population to a high-risk ESRD patient population without additional data.

While there is considerable variability in study design, the HEART and TIMI scores have been shown to correlate with angiographic CAD [13-21]. To the best of our knowledge, only a few studies have evaluated the predictive value of the HEART score for obstructive CAD, with variable results [14,27,28]. However, the TIMI score predictive value has been more extensively studied [13-21]. Our findings closely align with those arising from the general population admitted to the hospital with non-ST segment elevation acute coronary syndrome in terms of AUC [13,16,28].

However, given the variability of definitions of obstructive CAD, inclusion criteria, and methods among studies, comparing the optimal TIMI score cutoffs from our study to those of the general population proved challenging. While a few studies have deemed that a TIMI score >4 has been associated with single vessel obstructive CAD [18] and a TIMI score ≤4 with normal or non-obstructive CAD [19], this score cutoff was not internally validated by computing a receiver operating characteristic or C-statistic. Interestingly, one study found a TIMI score >2 to be the optimal score cutoff in the general population; however, differences in the definition of obstructive CAD and exclusion of patients with a proven non-cardiac etiology of chest pain may explain the lower cutoff score [16]. This study also did not specify patients with ESRD.

To our knowledge, our study is the first to identify an optimal cutoff in predicting severe CAD as defined by an anatomic lesion >70% stenosis in a specific, medically complex population. However, the study has limitations. This retrospective study was done at a single center, safety-net hospital, hindering the generalizability of its findings. However, the patients at safety-net hospitals have been shown to have underutilization of invasive coronary angiography and interventions, as well as worse outcomes, compared to patients at non-safety-net hospitals presenting with an acute myocardial infarction [29]. Therefore, our findings may be more applicable to similar facilities. Additionally, the small sample size may limit statistical power. Data collection through retrospective chart review relied on the characterization of the patient's presentation by the treating physician (for example, angina pectoris). Moreover, we did not stratify the severity of CAD by the number of affected coronary arteries or involvement of the left main coronary artery. We also strictly used coronary angiography and did not consider findings from other diagnostic tools, such as computerized tomography. Finally, we did not assess for long-term outcomes which have been reported with variable results [8]. It is noteworthy that, as mentioned above, the subset of patients with end-stage renal disease is subject to a higher risk of coronary events than the general population, regardless of the present angiographic findings. Thus, we emphasize the need for secondary prevention, including guideline-directed dietary, lifestyle, and pharmacologic recommendations.

## Conclusions

Our study establishes that, as previously described for the general population, a HEART score between six and seven, and a TIMI score between three and four adequately predict the presence of severe CAD in the ESRD patient population, presumably at higher risk for that outcome. These findings may help clinicians select the right candidates for coronary angiogram, which is especially relevant in areas with limited resources.
